# An active microbiome in Old Faithful geyser

**DOI:** 10.1093/pnasnexus/pgad066

**Published:** 2023-03-04

**Authors:** Lisa M Keller, Daniel R Colman, Eric S Boyd

**Affiliations:** Department of Microbiology and Cell Biology, 109 Lewis Hall, Montana State University, Bozeman, MT 59717, USA; Department of Microbiology and Cell Biology, 109 Lewis Hall, Montana State University, Bozeman, MT 59717, USA; Department of Microbiology and Cell Biology, 109 Lewis Hall, Montana State University, Bozeman, MT 59717, USA

**Keywords:** subsurface, hydrothermal, *Thermocrinis*, chemoautotrophy, Enceladus, Europa

## Abstract

Natural thermal geysers are hot springs that periodically erupt liquid water, steam, and gas. They are found in only a few locations worldwide, with nearly half located in Yellowstone National Park (YNP). Old Faithful geyser (OFG) is the most iconic in YNP and attracts millions of visitors annually. Despite extensive geophysical and hydrological study of geysers, including OFG, far less is known of the microbiology of geysed waters. Here, we report geochemical and microbiological data from geysed vent water and vent water that collects in a splash pool adjacent to OFG during eruptions. Both waters contained microbial cells, and radiotracer studies showed that they fixed carbon dioxide (CO_2_) when incubated at 70°C and 90°C. Shorter lag times in CO_2_ fixation activity were observed in vent and splash pool waters incubated at 90°C than 70°C, suggesting cells are better adapted or acclimated to temperatures like those in the OFG vent (∼92–93°C). 16S rDNA and metagenomic sequence data indicated that both communities are dominated by the autotroph *Thermocrinis*, which likely fuels productivity through the aerobic oxidation of sulfide/thiosulfate in erupted waters or steam. Dominant OFG populations, including *Thermocrinis* and subdominant *Thermus* and *Pyrobaculum* strains, exhibited high-strain level genomic diversity (putative ecotypes) relative to populations from nongeysing YNP hot springs that is attributed to the temporal chemical and temperature dynamics caused by eruptions. These findings show that OFG is habitable and that its eruption dynamics promote genomic diversity, while highlighting the need to further research the extent of life in geyser systems such as OFG.

**Significance Statement**

Natural thermal geysers are rare and are concentrated in several discrete locations on Earth and other planetary objects, like Enceladus and Europa. While the microbiology of hot springs is well studied, it remains unknown if waters erupted from geysers host microbial life. Geysers, like hot springs, contain all components necessary for life, including water (liquid and steam), CO_2_, and chemical disequilibria. Here, we show that geysed waters from OFG in YNP contain an active microbiome founded on autotrophy and chemical energy. Genomic data suggest a role for geyser dynamics in generating and/or maintaining genomic biodiversity. These findings further our understanding of the habitability of geysers and showcase potential approaches to detect life in similar systems on other planetary bodies.

## Introduction

Geysers are hot springs that periodically erupt liquid water, steam, and gas (1). There are fewer than 1,000 naturally occurring geysers worldwide due to the unique combination of factors needed for a geyser to form (2). All hot springs require a source of water, heat, and a permeable rock stratum overlying the heat source (3). An additional requirement to form a geyser is a plumbing system that has an appropriate structure to permit periodic fluid discharge (4, 5). Most geyser fields are hosted in rhyolitic volcanic terrains at high elevation that receive increased precipitation (4). Rhyolite is porous, allowing for infiltration of meteoric water that can then be heated by a magmatic source to form shallow hydrothermal aquifers (4). A buildup of pressure in these shallow aquifers due to rapid ascension, decompressional boiling of hydrothermal fluids, and subsequent buildup and release of volcanic gases can lead to periodic eruptions (6). Specifically, the buildup of dissolved carbon dioxide (CO_2_), which lowers the boiling point of water, has been suggested to be a key factor driving geyser eruptions (4, 7). Thus, although many continental volcanic hydrothermal systems exist globally, geysers are primarily restricted to areas meeting these specific requirements, and particularly in locations with rhyolitic volcanic bedrock.

Yellowstone National Park (YNP), USA, is located at an average elevation of 2,300 m and is heated by a shallow volcanic rhyolite flow that forms the world's largest subaerial continental hydrothermal system, hosting nearly half of the geysers worldwide (2, 8). This includes arguably the world's most iconic and well-studied geyser, Old Faithful geyser (OFG), which attracts millions of visitors annually (4, 9). OFG is a cone geyser located in the Upper Geyser Basin of YNP, an area characterized by neutral to alkaline waters rich in dissolved silica and chloride (i.e. alkaline-chloride waters) (10). After an eruption and subsequent cooling of erupted waters, silica sinter precipitates from water and this process, following hundreds to thousands of years of repeated cycles, formed the several meter-tall vertical cone of OFG (11). Further, erupted waters collect in several sinter-encrusted splash pools near the vent of OFG (9). Eruptive activity at OFG is predictable, occurring every 91–93 min, on average, permitting its safe investigation. Although thermal geysers such as OFG have been extensively studied from geological, geochemical, and hydrological perspectives (4, 9, 12–18), to the authors’ knowledge, there have not been any studies of the microbiology of geysed waters originating from the subterranean aquifers that source thermal geyser systems.

The temperature of the shallow aquifers that source thermal geysers is near boiling and, in the case of OFG, is ∼118°C at a depth of 21.7 m (14) (boiling at the elevation of OFG is ∼93°C), although steam condensates in the conduit have been measured at lower temperatures (e.g. 92–93°C) (14). The high temperature and lack of light in OFG likely restrict photosynthesis, which has an empirically observed upper temperature limit of ∼70°C (19, 20). Therefore, any microbial life in OFG is likely dependent on chemical forms of energy since the waters are topographically isolated from near surface runoff and the input of allochthonous organic matter. As such, microbial communities within OFG (and other geysers with similar characteristics) should be dominated by chemoautotrophic microbial populations that are likely to be supported by the near-continuous supply of water (or condensed steam), extensive disequilibrium in electron donors/acceptors such as H_2_S, H_2_, and O_2_ (21), and abundant carbon in the form of CO_2_ (22). In potential support of this hypothesis, waters that geyse from Crystal Geyser, which was formed anthropogenically, were shown to harbor an abundance of chemoautotrophs, suggesting that the subsurface aquifers sourcing this cold water geyser are inhabited by organisms dependent on chemical energy (23).

Here, we investigated the abundance, activity, and composition of microbial communities supported by OFG thermal fluids with the goal of establishing whether geysed waters exhibited evidence for microbial communities and measurable activity indicative of the presence of an active subsurface hydrothermal biosphere. Samples of erupted vent water and erupted water that collects in a splash pool adjacent to the vent were sampled and compared. We hypothesized that OFG hosts active cells that are supported by substrates supplied by volcanic degassing and/or water rock interaction. Further, we hypothesized that the large temporal temperature gradient in the splash pools, due to the periodic input of geysed water, selects for increased microbial diversity. These hypotheses were evaluated via microscopy, metabolic activity assays, and targeted 16S rRNA gene and metagenomic sequencing, respectively. The results point to the presence of microbial populations in erupted waters that are specifically adapted to the temperatures (∼92–93°C) of the vent of OFG. The results are discussed as they relate to the habitability of geyser systems and the role of temporal geysing dynamics in the generation and maintenance of microbial biodiversity. The results also suggest several potential approaches that could be used to detect life in eruptive plumes from geyser systems on other planets where they have been detected.

## Results and discussion

### Site description

Eruptions of OFG are predictable over short time scales, a feature that has enabled its continued and safe scientific study for nearly a century (8). While the eruption interval is predictable on the short term, it has fluctuated substantively over longer time periods, ranging from eruption intervals averaging 65 min in 1883, 65–75 min in 1911, 64 min in 1948, 80 min in 1979 to 91 min in 2011 (8, 15, 24, 25). These changes are associated with alteration of the plumbing and recharge system sourcing OFG due to major earthquakes, although some changes are attributable to climatic variation, such as variation in the amount of precipitation from preceding seasons, recharge during glacial-interglacial periods, or changes in gravity due to Earth's tidal forces and barometric pressure (11–13, 26). Unlike intervals between eruptions, eruption duration has been nearly constant since the first measurements in 1883 (15, 24, 25). OFG's eruption duration currently varies from 2.0 to 5.0 min. However, recent data from dating trees silicified in the cone of OFG suggests that the eruptive dynamics and duration were likely significantly different during drier climate periods in the 19th century (11).

The cone of OFG is located above the surrounding area on siliceous sinter, or geyserite (Fig. S1). Steam temperatures within the conduit of OFG range from 92 to 93°C between eruptions, and water column temperatures range depending on the depth of measurement and the time the measurement is taken (14, 16). The temperature at the top of the water column (∼22 m below cone) is usually above boiling (110–118°C) at the elevation of OFG (boiling point of 93°C at 2,300 m), although it can be much higher (130°C) when upwelling from the hydrothermal aquifer occurs as an eruption nears (14, 16). Like many of the alkaline-chloride waters in the Upper Geyser Basin hot springs (27, 28), the pH of waters collected from the pool and the vent in 2018, 2019, and 2020 ranged from 8.7 to 9.0 (Table 1), similar to previous measurements of OFG waters (27–29). The pH of the aquifer sourcing OFG is thought to be near neutral, with the pH of erupted waters being slightly higher due to CO_2_ degassing (14). OFG waters also had high chloride (Cl^−^) and low sulfate (SO_4_^2−^) concentrations (Table 1 and Fig. S2A), similar to other alkaline-chloride waters in hot springs of the Upper Geyser Basin and previous measurements of OFG (29, 30). The high Cl^−^ and low SO_4_^2−^ concentrations suggest that the waters sourcing OFG come from the deep hydrothermal aquifer (Fig. S2A). This is consistent with the minimal amount of tritium (half-life of 12.32 ± 0.02 years) previously measured in the outflow channel waters of OFG (29), suggesting a relatively long recharge time for OFG waters. Indeed, nearly 24 eruptions were needed to dilute Rhodamine B dye that was added to subsurface OFG waters to levels that were below detection (31). In addition, SO_4_^2−^ concentrations of the OFG waters (8–11 mg L^−1^) indicate that these waters likely underwent phase separation deep in the hydrothermal system and/or that sulfate precipitated from these waters as anhydrite (CaSO_4_) due to boiling (10, 30) (Table 1 and Fig. S2A).

**Table 1.** Geochemical data collected for water erupted/geysed from Old Faithful geyser (vent) and from the splash pool (pool) adjacent to the vent in 2018, 2019, and 2020. Erupted waters were not sampled in 2018. The concentration of ferrous iron in all years was below the limit of detection (∼5 µM) in all samples and is thus not reported. Dissolved organic carbon (DOC) values were below the limit of detection (<0.12 mg L^−1^) in vent and pool samples in 2018 and 2019 and are thus not reported. DOC was not measured in 2020. The temperature of the vent waters is not reported since it could not be safely measured during the eruption. Additional geochemical data is reported in Table S6.

Site	Year	Temp (˚C)	pH	Conductivity (mS cm^−1^)	Sulfide (mg L^−1^)	Sulfate (mg L^−1^)	Chloride (mg L^−1^)
**Vent**	2020	N/A	8.9	2.8	0.56	7.7	533
**Pool**	2020	70.5	8.7	3.6	0.56	8.3	520
**Vent**	2019	N/A	9.0	2.5	0.54	8.0	493
**Pool**	2019	70.8	8.9	3.7	0.41	8.0	507
**Pool**	2018	73.0	9.0	3.8	0.61	11	400

**Fig. 1.**
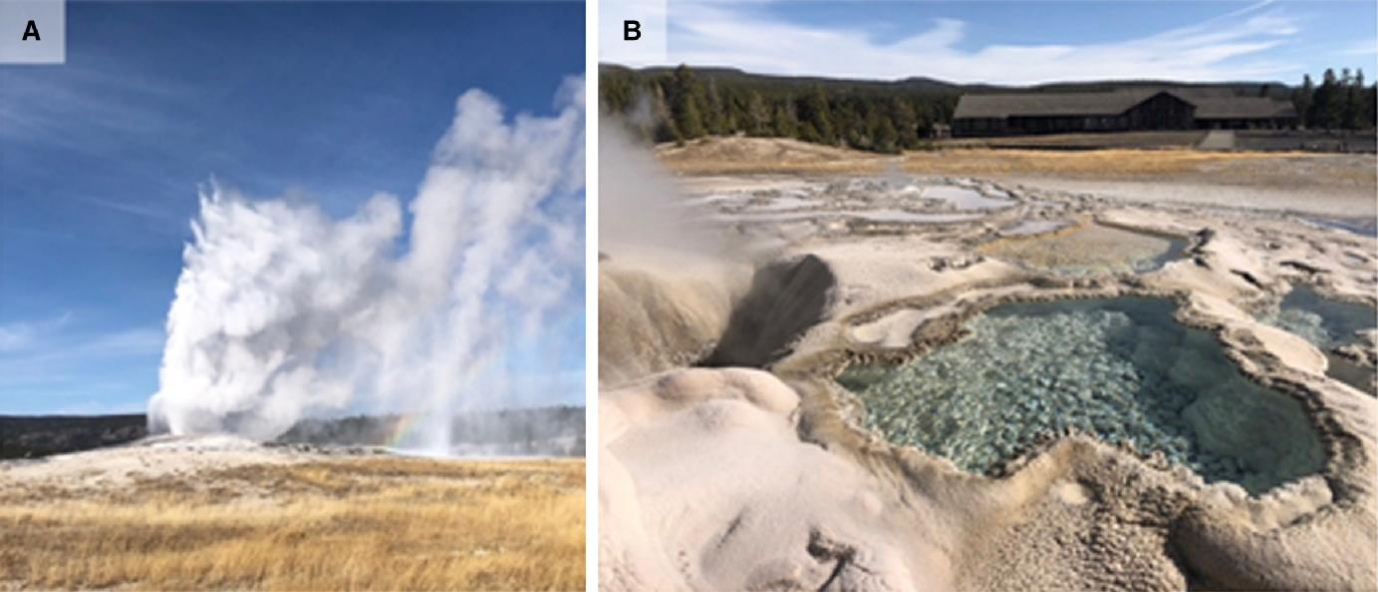
Old Faithful geyser (OFG) vent and splash pools investigated in this study. A) Image of OFG erupting prior to sampling. B) Image of the splash pool where the samples were collected beside the OFG vent (seen to the left of the pool).

OFG, and other geysers like it, often form pools adjacent to their vents as geysed waters cool that are saturated with silica, allowing the silica to precipitate (9) (Fig. 1B). The aquifer supplying water to OFG is saturated with silica due to the dissolution of rhyolite in the subsurface at high temperature, that then precipitates on the surface of the cone as the water cools following an eruption (17, 32–35). Continual deposition of silica with each eruption cycle resulted in the formation of the cone and the pools adjacent to the cone (Fig. 1) that do not have an obvious source of water other than the water erupting out of OFG or water from meteoric precipitation. To evaluate if the waters in these pools were representative of OFG eruption vent waters, geochemical data were collected from the primary pool adjacent to the cone (pool waters) and compared with water geysed from the OFG vent during an eruption that was collected in a presterilized bin (vent waters). The conductivity values of waters in the pool (3.6–3.8 mS cm^−1^) were higher than those in the vent waters (2.5–2.8 mS cm^−1^), regardless of the sampling year (Table 1). This indicates that pool waters are likely sourced primarily from erupted waters rather than meteoric water and that the pool waters have undergone boiling and evaporation that concentrated solutes. This is supported by ratios of Cl^−^ to SO_4_^2−^ (two primary anions often used to identify sources of fluids to hot springs [30, 36]) that were similar among pool and vent waters. Likewise, OFG waters sampled from the pool in 2019 and 2020 were depleted in δ^2^H and δ^18^O relative to vent waters (Fig. S2B and C). The slope of the relationship between δ^18^O plotted as a function of δ^2^H for OFG pool versus vent waters was ∼4, similar to the slope (∼3) for thermal waters in YNP that have undergone boiling and evaporation (30).

**Fig. 2.**
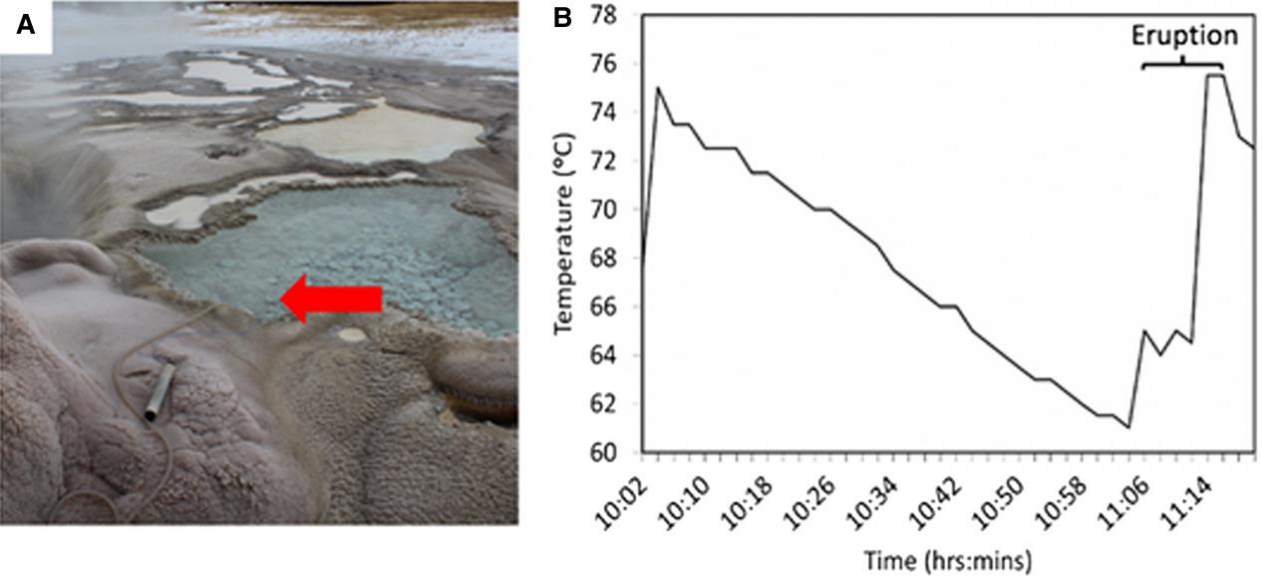
Fluctuations in the temperature of waters in the splash pool adjacent to the vent of Old Faithful geyser during an eruption cycle. A) Image showing the location of the thermocouple in the splash pool prior to an eruption (red arrow shows the placement of the thermocouple). B) Temperature of the splash pool logged from the end of one eruption through the entirety of another eruption.

To further examine the extent to which the vent waters impact the waters in the splash pool, a thermocouple was placed in the pool on 2018 November 9, for the duration of an eruption cycle (Fig. 2A). Once the thermocouple adjusted to the temperature of the pool, the temperature of waters steadily decreased by roughly 0.23°C min^−1^ from 75°C at 10:04 AM to 61°C at 11:04 AM (Fig. 2B). After the steady decrease, a rapid increase in temperature (1.10°C min^−1^) was observed, followed by a temperature decrease over a few minutes, coinciding with the commencement of eruptive activity. OFG's water level often begins to oscillate prior to erupting, causing small eruptions of water to occur prior to the main eruption event (15, 18, 37). These oscillations reach a critical depth (∼10 m) in the subsurface, increasing the pressure in the system and causing a cascading effect that triggers the main eruption (15, 18). This is evident in the temperature data (Fig. 2B), where a much larger increase in temperature was observed after the initial increase. Together with the geochemical data, these observations suggest that the water in the pools adjacent to the OFG vent is sourced and impacted by OFG's eruption cycle.

To evaluate the uniqueness of the geochemistry and microbial biodiversity in OFG relative to other geysers of the region, Spouter Geyser (SG), Black Sand Basin, and Upper Geyser Basin were also sampled. The SG eruption interval is less predictable (∼1.5 h) than that of OFG, and consequently, a small pool was sampled with a water reservoir that fills during the SG eruption cycle (SG high) and then partially drains (SG low) between eruptions. Water for geochemical analyses was collected during both high and low-water level phases, although samples for DNA extraction and metagenomic sequencing were collected over a period that spanned both phases. SG water temperatures fluctuated between 91 and 81°C during and following an eruption, whereas the pH of the water varied minimally from 8.6 to 8.5 during and following an eruption. SG conductivity values were 5.0 mS regardless of the time of sampling. Finally, SO_4_^2−^ and Cl^−^ concentrations were 11 and 280 mg L^−1^, respectively, regardless of sampling time (Table S1). These values are consistent with this geyser being sourced by the parent hydrothermal aquifer (30, 33) and its eruptive activity affecting the temperature of the sampled SG pool, but its geochemistry to a lesser extent.

### Abundance and morphology of microbial cells in OFG waters

To assess the abundance and morphology of cells in the OFG vent and pool waters, fluorescent microscopy of samples fixed on site was performed. A mix of cell morphologies including cocci, rods, and filamentous microorganisms was observed in both vent and pool water samples (Fig. S3). The concentrations of cells were surprisingly similar, with the pool water hosting 6.02 ± 0.63 × 10^4^ cells mL^−1^, and the vent water hosted 3.54 ± 0.19 × 10^4^ cells mL^−1^, despite the lower average temperature of the pool (∼70°C) water when compared with the vent of OFG (∼92–93°C) or the water column below (>110°C). While the abundance of cells might be expected to decrease with increasing temperature due to diversion of metabolic energy to meet increasing demands associated with maintenance of biomolecules at high temperature (38), it is possible that the dynamic (fluctuating) nature of the pool environment limits biomass (cell) production in this ecosystem. Nonetheless, while there is limited reported cell count data from YNP, in particular in circumneutral to alkaline springs, these values are among the lowest measured for continental subsurface fluids globally (39), suggesting that they represent the lower limit of microbial productivity in subsurface-derived hydrothermal fluids.

### Chemoautotrophic primary production of OFG microbial communities

**Fig. 3.**
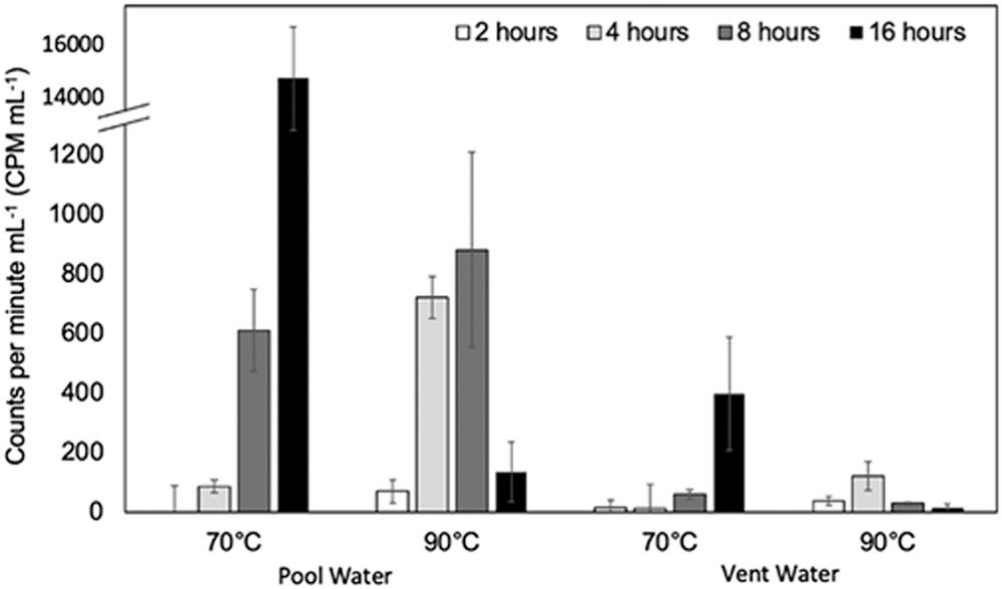
Assimilation of ^14^C-bicarbonate in counts per minute (CPM) by planktonic populations in biotic microcosms containing waters from the splash pool (pool) or erupted/geysed waters (vent). Triplicate microcosms were incubated for 2, 4, 8, and 16 h in the dark at the specified temperatures. The break in the *Y*-axis scale was introduced to allow for visualization of the considerably higher CPMs measured in microcosms containing splash pool waters incubated at 70°C for 16 h.

To begin to assess whether cells observed in pool and vent waters were viable and active, ex situ activity assays measuring light-independent fixation of ^14^C-bicarbonate to biomass were conducted. Activities were measured for planktonic cells collected from both pool and vent waters in microcosms incubated at both 70 and 90°C to evaluate the extent to which chemoautotrophic populations present in each water type were adapted to the prevailing thermal regime of the pool (∼70°C; see Fig. 2B), the vent (∼92–93°C), or possibly to both thermal regimes (Fig. 3).

In microcosms containing vent waters, ^14^CO_2_ assimilation activity attributable to microbial cells (biotic minus abiotic controls) was detected at 90°C after only 2 h of incubation. Activity increased up to 4 h of incubation, followed by decreased activity. The decrease at later time points likely indicates cell lysis or turnover of fixed carbon into a form (e.g. chemosynthate) that passes through the 0.2 µm filters that were used to concentrate biomass following the termination of microcosm assays. In contrast, activity attributable to cells from vent waters incubated at 70°C was only detected in microcosms following 8 h of incubation, with activity subsequently increasing until 16 h of incubation. These differing responses in autotrophic activity suggest that at least two populations of chemoautotrophs were present in the vent waters. Alternatively, a single autotrophic population could have been present in the vent water that is better adapted to 90 than 70°C, resulting in a substantial lag phase in detectable ^14^HCO_3_^−^ assimilation in the latter condition (Fig. 3). It has been suggested that the duration of the lag phase is a proxy for the extent of adaptation by an organism (or a community of organisms) to use a given substrate (40–42), with shorter lag times indicative of a greater extent of adaptation. To this end, it is likely that at least one population of autotrophic cells in OFG vent waters was adapted (or better acclimated at the time of sampling) to 90°C, near to the temperature of OFG vent between eruptions (∼92–93°C) (14, 16). This observation suggests the presence of an active microbiome in the vent, since a population of thermophilic cells would be difficult to sustain through repeated eruptions unless they are active and replicating when the vent is at ∼92–93°C. Notably, this is the temperature range that is most commonly encountered in the vent conduit, except when OFG is actively erupting (14).

In microcosms containing pool water, ^14^CO_2_ fixation activity attributable to microorganisms was detected at 70°C following 4 h of incubation and continually increased until 16 h of incubation. In pool water microcosms incubated at 90°C, activity was detectable after 2 h of incubation. Activity increased between 2 and 4 h of incubation, did not change significantly between 4 and 8 h, and then decreased between 8 and 16 h of incubation. Thus, the overall rate of ^14^CO_2_ fixation activity was significantly higher in microcosms incubated at 70°C than at 90°C, regardless of whether the waters were derived from the pool or vent (Fig. 3). Nevertheless, differences in lag times prior to commencement of autotrophic activity in microcosms incubated at 70 and 90°C, the higher overall rate of autotrophic activity at 70 than 90°C, and the timing of decrease in biomass-fixed ^14^CO_2_ in microcosms containing vent and pool waters suggest the presence of at least two autotrophic populations in both the pool and vent water that are adapted to different thermal regimes. Like vent waters, the shorter lag phase for commencement of CO_2_ fixation activity suggests that at least one population of autotrophic cells in OFG waters is better adapted (or better acclimated) to grow at 90°C. These observations are consistent with the waters sourcing the splash pool deriving from the erupted waters from the OFG vent. It should be noted that temperature differences and CO_2_ degassing dynamics render it difficult to make direct quantitative comparisons of ^14^CO_2_ incorporation between pool and vent water communities (see Materials and methods for additional details).

### Taxonomic composition of OFG microbial communities

**Fig. 4.**
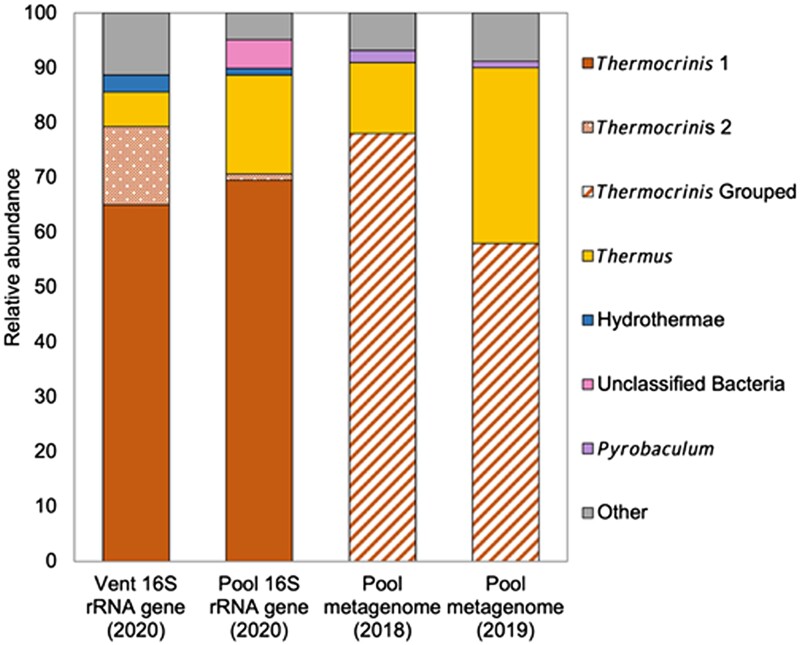
Taxonomic composition of PCR-amplified 16S rRNA genes and metagenomic sequence from Old Faithful geyser splash pool (pool) and erupted/geysed (vent) water communities. Taxonomic data are reported for 16S rRNA gene operational taxonomic units (OTUs) defined at 97.0% identities to the highest rank possible based on affiliation to known cultivars. Organisms that could not be classified beyond the specified rank are depicted with the heading “unclassified.” OTUs that represented <0.1% of the relative abundance of a given community were binned together and are depicted as “other.” A full description of the taxonomy of 16S rRNA gene OTUs and metagenome-assembled genomes (MAGs) is reported in Table S2 and Table S4, respectively. For metagenomic sequences, the relative abundance of *Thermocrinis*, *Thermus*, and *Pyrobaculum* was evaluated based on bin-independent read mapping against the Kraken2 database. “*Thermocrinis* Grouped” represents all mapped *Thermocrinis* reads in the 2018 and 2019 metagenomes and thus does not delineate *Thermocrinis* subtypes. Since the 16S rRNA gene amplicon data are normalized to total sequences obtained (relative abundance) or total sequences for metagenomic sequence (binned and unbinned), these data have been included on the same plot to facilitate comparison.

To identify putative chemoautotrophic microorganisms in pool and vent water communities, 16S rRNA genes were amplified and sequenced from DNA collected from planktonic biomass. Over 70% of the 16S rRNA genes recovered from the vent and pool water communities were affiliated with the genus *Thermocrinis* (Fig. 4 and Table S2). All cultured *Thermocrinis* strains are capable of fixing CO_2_, although most strains are also facultatively chemoorganotrophic (43, 44). Moreover, culture-independent studies of hot springs have revealed that *Thermocrinis* tend to be the dominant chemoautotrophs in the highest temperature alkaline-chloride springs in YNP (45–48). Thus, the dominance of *Thermocrinis* in both the vent and pool waters is consistent with their inferred role as primary producers in circumneutral to alkaline continental hot spring systems globally (49, 50). Two predominant 16S rRNA gene operational taxonomic units (OTUs; >1.0% relative abundance) affiliated with *Thermocrinis* were present in the waters, one of which (OTU001) was most closely related (99.2% 16S rRNA gene identity) to *Thermocrinis ruber* strain OC 1/4 (*Thermocrinis* 1 in Fig. 4) and the other of which (OTU005) was most closely related to 16S rRNA genes from an uncultured *Thermocrinis* group recovered from several YNP hot springs that include Joseph's Coat Hot Spring, Perpetual Spouter, Evening Primrose, Octopus Spring, Bechler Spring, an unnamed spring in the Gibbon Geyser Basin (GGB063 (48)), an unnamed spring in Norris Geyser Basin, Roadside West, and Roadside North (*Thermocrinis* 2 in Fig. 4) (Fig. S4). The uncultured *Thermocrinis* 2 OTU was more abundant in the vent planktonic community, whereas the *T. ruber* OTU exhibited similar abundances in the vent and pool water communities (Fig. 4). *Thermocrinis ruber* strain OC 1/4 was originally isolated from the outflow channel (82–88°C) of Octopus Spring, an alkaline-chloride hot spring in the Lower Geyser Basin of YNP (50).

Like other *Thermocrinis* (51), *T. ruber* grows chemolithoautotrophically with hydrogen, thiosulfate, or elemental sulfur as electron donors and oxygen as the electron acceptor. More recent data indicate that this species can also oxidize arsenite and monothioarsenate (52). The uncultured *Thermocrinis* 2 group has been detected in DNA extracted from communities collected from several alkaline-chloride YNP springs, including “Bison Pool” (47) (Table S3). A prior genome reconstruction of one member of the uncultured group (Oct-r1) revealed a similar metabolic capacity as *T. ruber*, except without the apparent capacity to oxidize hydrogen (45). In addition to the predominant *Thermocrinis* phylotypes, >5,500 other low-abundance (<1.0%) OTUs in OFG waters were classified as *Thermocrinis*, indicating the presence of considerable intraspecific genetic diversity among the *Thermocrinis* population. Thus, multiple, phylogenetically distinct, autotrophic *Thermocrinis* strains were present in OFG waters that belong to different groups that have been recovered primarily from YNP springs, but from springs with somewhat distinct pH and temperature profiles (Fig. S4 and Table S3). Consequently, the differing chemoautotrophic activities at different temperatures observed in OFG waters could be due to the activities of several *Thermocrinis* strains with different temperature optima.

In addition to the two *Thermocrinis* strains, both communities contained 16S rRNA gene OTUs closely related to the heterotrophic genus *Thermus* (53), putatively facultatively autotrophic genus *Pyrobaculum* (43), putatively heterotrophic organisms only classifiable to the phylum level of Hydrothermae (46), and several unclassifiable bacterial OTUs and numerous low-abundance OTUs. As a result of the vent and pool being at a higher elevation than the surrounding landscape, the input of allochthonous organic carbon is likely minimal, thereby selecting for dominant autotrophic populations of *Thermocrinis*. Lysis of *Thermocrinis* cells or cross-feeding of organic carbon from *Thermocrinis* populations to the heterotrophic *Thermus* and other lower abundance heterotrophic populations, and subsequent oxidation of that organic carbon, may be responsible for community productivity and the decrease in fixed CO_2_ observed in ^14^C-uptake microcosm assays incubated at 90°C.

To evaluate the functional potential of populations within the OFG communities, metagenomic sequences were obtained for 2018 and 2019 communities from filtered pool waters. Due to low DNA concentrations from vent waters, the preparation of vent water metagenomic DNA libraries failed and thus were not sequenced. Nonetheless, the taxonomic composition of pool water-assembled contigs was similar to that of both the pool and vent water PCR-amplified 16S rRNA genes. Specifically, *Thermocrinis* populations dominated the metagenomic data and comprised 78 and 58% of the mapped reads for both the 2018 and 2019 metagenomes, respectively, like the PCR-amplified 16S rRNA gene abundances from 2020. *Thermus* was the second most abundant population based on metagenomic sequence data from 2018 and 2019 (13 and 32%, respectively), like their abundances based on PCR-amplified 16S rRNA gene data from 2020 (Fig. 4). In addition, *Pyrobaculum* comprised <0.1% of the PCR-amplified 16S rRNA gene reads compared with about 1% of the mapped metagenomic reads. This difference could be attributed to biases associated with the primers used in amplifying 16S rRNA genes in 2020, or differences in the abundances of minor community members between years.

Assembly of pool water metagenomes resulted in the recovery of multiple highly heterogenous and incomplete *Thermocrinis-*affiliated metagenome-assembled genomes (MAGs) that dominated the 2018 and 2019 metagenomes. Attempts to produce MAG representatives of individual populations (and not mixtures of multiple, closely related populations) were made via multiple assembly/assembler strategies, multiple binning strategies, and/or the use of reference-based assemblies. The inability to produce higher quality *Thermocrinis* MAGs points to the presence of multiple, highly related strain-level variants in these waters, consistent with the abundance of numerous *Thermocrinis* 16S rRNA gene OTUs, and genomic data (discussed below). Coverages of reads (estimated by mapping to the *T. ruber* genome) were 1,582 and 3,224× for the 2018 and 2019 metagenomes, respectively, indicating that additional short read sequencing would be unlikely to resolve strain-level variants into individual genotypes. Unfortunately, the recovery of insufficient amounts of DNA from pool waters precluded the generation of long insert libraries needed to generate longer reads (e.g. via the PacBio platform), which may have been able to further resolve variants into independent genotypes. Nonetheless, the two dominant 16S rRNA gene phylotypes identified in the PCR-based survey in 2020 were present in the metagenomes from 2018 and 2019, thus supporting the hypothesis of multiple *Thermocrinis* populations that stably existed in OFG waters and that likely occupied different niches (e.g. different growth optima).

Strain-level variants, or putative ecotypes (i.e. a genetically cohesive population that occupies a slightly different ecological niche as its ancestor (54)), can be generated and maintained in hot spring environments that have partially overlapping niches, either temporally or spatially (55–59). In hot springs, such ecotype variation has been observed among *Synechococcus* strains that have different temperature optima and/or light absorbance properties, allowing for their stable coexistence (55–57, 60). Consequently, it is possible that the temporal dynamics of OFG and SG, in particular the splash pool of OFG (Fig. 2B) and the fluctuating pool adjacent to SG where strain-level diversity was detected, create multiple thermal (and/or possibly chemical) niches that select for ecotypes with differing temperature optima (and/or differing affinities for chemical nutrient substrates).

To begin to assess this possibility, the genetic diversity of *Thermocrinis* in the OFG pool was further investigated by measuring the extent of intraspecific genomic (nucleotide) diversity within *T. ruber*–associated metagenomic reads (>95% ANI to the *T. ruber* genome) in the OFG pool and the nearby SG geyser. Further, these data were compared against that from ten other available nongeysing YNP hot spring metagenomes generated from high-resolution short-read shotgun metagenomic analyses of waters and sediments sequenced at a similar depth as the OFG and SG metagenomes (Figs. S3 and 5). Publicly available metagenomes from biofilm/filament-based communities (including from Bison Pool, Octopus Spring, and Bechler Spring (61, 62)) were not included in these analyses to avoid conflation of population dynamics due to nonequivalent comparisons of biofilm populations to water/sediment populations. In addition, these biofilm metagenomes were sequenced to comparatively low depths using Sanger sequencing, thereby preventing the high-resolution read mapping necessary to perform downstream genomic diversity calculations.

**Fig. 5.**
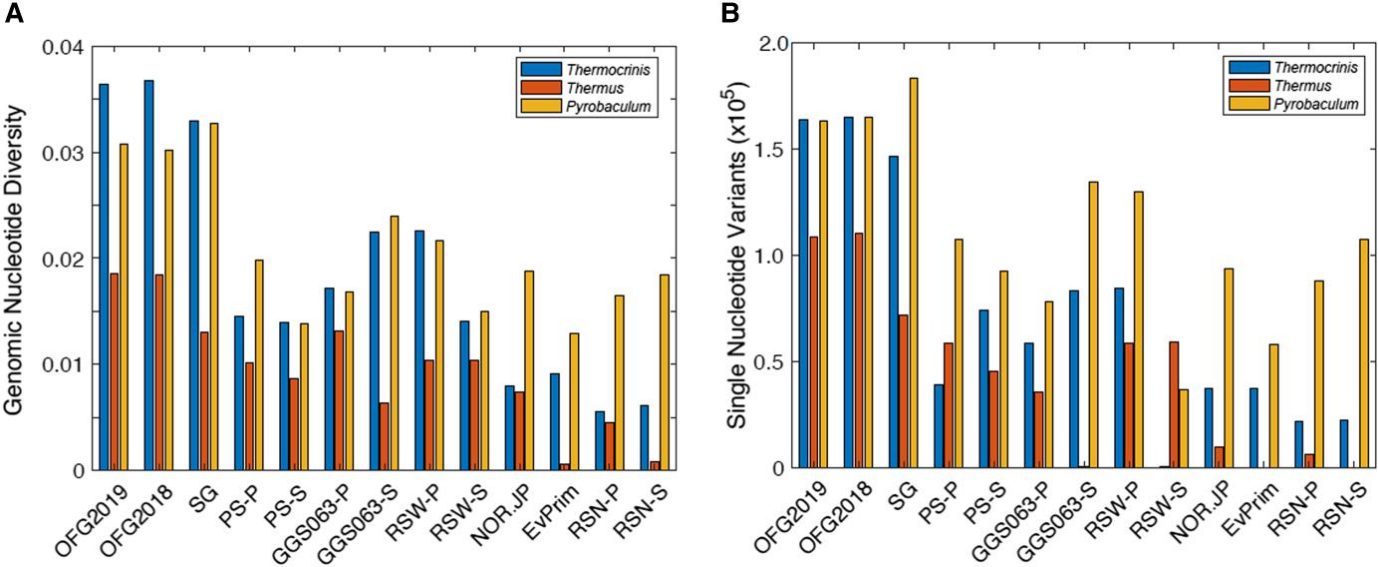
Genomic diversity associated with dominant populations in Old Faithful geyser (OFG2018, OFG2019) and Spouter Geyser (SG) communities compared with other communities from nongeysing Yellowstone National Park (YNP) hot springs (defined below). A) Rarified nucleotide diversity among reads mapped to the complete *Thermocrinis ruber*, *Thermus aquaticus*, or *Pyrobaculum yellowstonensis* reference genomes from OFG, SG, and YNP hot spring waters (P) or sediments (S). Reads were mapped to the respective genomes and filtered to include those with >95% (*Thermocrinis* and *Thermus*) or 90% (*Pyrobaculum*) ANI. Rarified nucleotide diversity calculations are shown to account for potential biases due to differences in read coverage among samples. Additional hot spring physicochemical data and metagenome statistics for those communities shown in the bar graph are reported in Table S3. B) SNV counts among the read sets mapped to *T. ruber*, *T. aquaticus*, and *P. yellowstonensis* genomes among OFG, SG, and YNP hot spring waters (P) or sediments (S). The same datasets and metagenomes used in panel A were also used here. Abbreviations for hot springs and references (if available) for descriptions of those springs are as follows: GGS063, Gibbon Geyser Basin Spring 63 (85.9°C, pH 7.1 (48)); PS, Perpetual Spouter (84.1°C, pH 7.4 (63)); RSW, Roadside West (68.5°C, pH 6.7 (64)); NOR-JP, unnamed spring in the Norris Geyser Basin (76.5°C, pH 6.2); EvPrim, Evening Primrose (77.4°C, pH 5.6 (65)); RSN, Roadside North (86.2°C, pH 5.2 (64)). Additional details for the metagenomes are provided in Table S3.

The 2018 OFG pool and 2019 OFG pool communities contained the highest level of *Thermocrinis*-associated nucleotide diversity (Fig. 5A), consistent with levels of single-nucleotide variants (SNVs; Fig. 5B) that were also highest in the OFG pool communities. The SG community contained nearly equivalent levels of *Thermocrinis* nucleotide diversity, while the remaining *Thermocrinis* populations from other metagenomes in nongeysing hot spring waters or sediments contained between 2- and 5-fold less nucleotide diversity than in the OFG pool. Likewise, the SG and OFG communities contained nearly equivalent levels of *Thermocrinis* SNVs, which would be expected to differentiate closely related strains or ecotypes (66). Like nucleotide diversity, the number of SNVs in SG and OFG *Thermocrinis* populations were 2- to 4-fold higher than nongeysing hot spring waters or sediments. Thus, the OFG pool water and the SG water communities contained uniquely high levels of *Thermocrinis* genomic diversity relative to other nongeysing hot springs in YNP.

To further investigate the extent of subspecies-level *Thermocrinis* diversity in the OFG, RNA polymerase subunit beta (RpoB) fragments were identified in the entire OFG pool metagenomic assembly (i.e. not just MAGs) and subjected to phylogenetic analysis, alongside *Thermocrinis* RpoB from public databases of isolates and metagenomes. Phylogenetic analysis revealed the presence of a YNP-specific group of *Thermocrinis* from OFG and SG that were related to the only *Thermocrinis* species previously isolated from YNP, *T. ruber* strain OC 1/4 (Fig. S5A). Intriguingly, the OFG pool community contained four distinct *Thermocrinis* RpoB phylotypes, while the SG assembly contained two distinct phylotypes. Read mapping of the 2018 and 2019 OFG metagenomes to the four *Thermocrinis* phylotype contigs revealed that all four were present in both the 2018 and 2019 OFG pool communities. Only one other nongeysing spring water metagenome included in the genomic diversity calculations (Evening Primrose; pH 5.6, 77.4°C (65)) contained more than one RpoB phylotype (Fig. S5A). Further, inclusion of *Thermocrinis* RpoB phylotype sequences from all publicly available YNP hot spring metagenomes revealed that the only communities harboring multiple phylotypes were those from filamentous biofilms collected from Bison Pool (62) and Bath hot springs (61) (Fig. S5A).

**Fig. 6.**
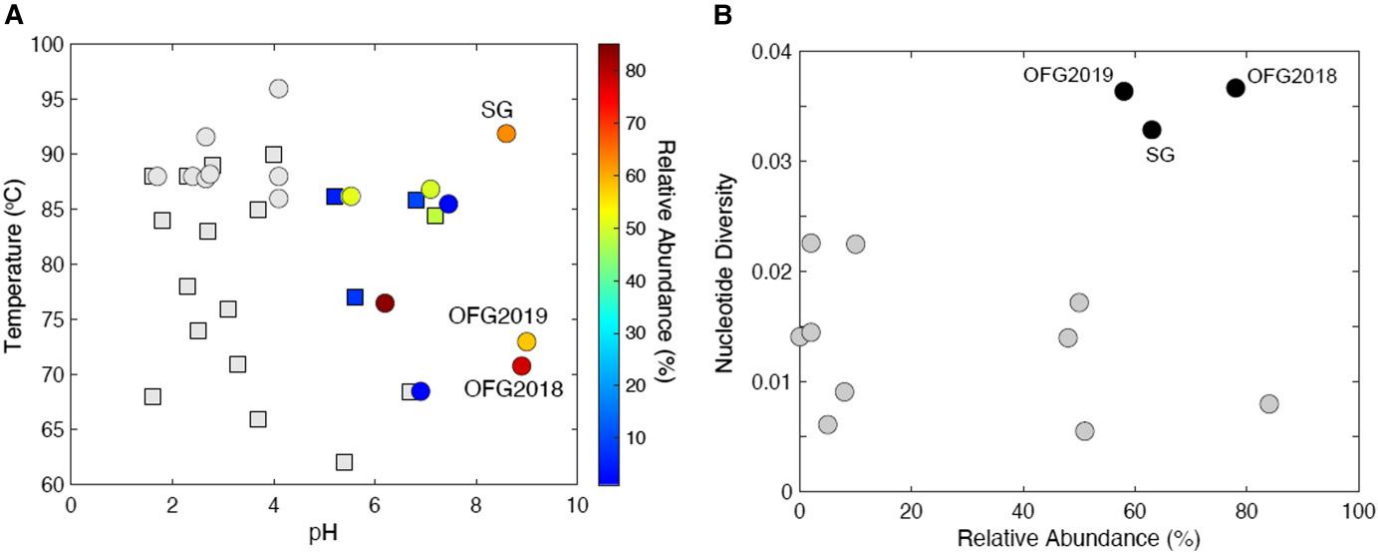
Distribution of *Thermocrinis* across Yellowstone National Park (YNP) hot spring and geyser communities and their association with genomic nucleotide diversity. A) Relative abundance of *Thermocrinis* populations in 35 hot spring and geyser communities (Table S3). Each point represents one metagenome that is plotted based on spring temperature and pH. Metagenomes with <1% of reads identified as *Thermocrinis* are shown as grey squares (sediment metagenomes) or circles (water metagenomes), while those with *Thermocrinis* populations comprising >1% of mapped reads are colored according to the scale to the right of the plot. B) Genomic nucleotide diversity of *Thermocrinis*-like populations (based on >95% ANI mapping to the *Thermocrinis ruber* genome) as a function of the relative abundance of *Thermocrinis* in those metagenomes. Nongeysing hot springs (*n* = 10) are shown as grey circles, and geysers are shown as black circles and are labeled as Old Faithful geyser (OFG2018, OFG2019) and Spouter Geyser (SG).

As described above, *Thermocrinis* are ubiquitous and abundant members of circumneutral to alkaline YNP hot spring communities based on previous cultivation-independent analyses (61, 67, 68). Nevertheless, nearly all of these previous studies were of biofilm streamer communities that are presumably generated by *Thermocrinis* themselves (68), while little is known of their distribution in nonbiofilm communities. A comparison of *Thermocrinis* relative abundances in sediment and water community metagenomes used in the genomic diversity analyses above, in addition to others generated in our other studies, confirmed their general dominance of hot spring communities at circumneutral to alkaline pH and high temperature (Fig. 6A and Table S3). Moreover, *Thermocrinis* genomic nucleotide diversity generally scaled inversely with relative abundance among nongeysing hot springs (Fig. 6B), as would be expected by the dominance of communities by a single successful ecotype. The notable exceptions to these trends were the relatively higher genomic nucleotide diversity levels of the OFG and SG geyser populations (Fig. 6B). Thus, these results point to elevated levels of *Thermocrinis* genomic and possibly ecotypic diversity within OFG and SG that could be related to the dynamic temperature fluctuations of these environments.

The second most abundant population in the metagenomic data for both the 2018 and 2019 metagenomes was related (96.5% ANI) to *Thermus aquaticus*. *Thermus aquaticus*–associated reads comprised 13 and 32% of the total reads from the 2018 and 2019 metagenomes, respectively. MAGs were also generated for *Thermus* but, like those for *Thermocrinis*, they were highly incomplete and heterogenous. Nevertheless, the RpoB sequence of the MAGs shared 100% amino acid identity to that of the obligate heterotroph, *Thermus aquaticus* (Fig. S5B), which was originally isolated from Mushroom Pool (62.9 to 72.9°C) in YNP (69). Like the *Thermocrinis* population, the *Thermus* MAGs from OFG and SG exhibited higher nucleotide diversity than those from nongeysing YNP hot springs (Fig. 5). The third most abundant population in both the 2018 and 2019 metagenomes was most closely affiliated with *Pyrobaculum* (96% ID to *Pyrobaculum yellowstonensis* WP30 based on RpoB sequences; Fig. S5C) that comprised 2.2 and 1.2% of the communities for 2018 and 2019, respectively. Like the *Thermocrinis* and *Thermus* populations, the *Pyrobaculum* population exhibited much higher nucleotide diversity than those from other nongeysing YNP hot springs (Fig. 5). The other MAGs that represented >1% of the communities in both 2018 and 2019 metagenomes were not classifiable past the class or order-level designation (Table S4). In contrast to the presence of multiple *Thermocrinis* and *Pyrobaculum* phylotypes based on RpoB sequences, multiple *Thermus* RpoB phylotypes were not observed in the OFG metagenomes (Fig. S5).

Together, these data point to a role of geyser temporal dynamics in the generation and maintenance of population-level biodiversity and possibly ecotypic biodiversity, particularly among the putative autotroph *Thermocrinis*. Consequently, it is suggested that multiple thermal (and possibly chemical) niches generated by temporal input of geysed fluids select for variants with differing temperature optima (or differing affinities for chemical nutrient substrates). While additional experimentation, such as culturing and establishing different temperature optima for strains, is needed to fully establish such a relationship, the data point to the dynamic nature of geysers in the generation of population-level variant biodiversity that is maintained in hydrothermal systems due to the periodic and often repeated nature of eruptive activity. Steam and spray from geysers could also promote dispersal of such strain-level variants to other hydrothermal features (hot springs, geysers, and fumaroles) that house unoccupied (or suboptimally occupied) niches that fit colonizer physiology.

### Functional composition of OFG microbial communities

The *Thermocrinis* MAGs from OFG were functionally characterized since they comprised multiple *Thermocrinis* strain-level variants (i.e. ecotypes; Fig. 4) that would be expected to exhibit minimal variation in their encoded functions (54). The *Thermocrinis* MAGs encode the rTCA cycle for CO_2_ fixation including the key enzymes, citryl-CoA synthetase and citryl-CoA lyase (Table S5). The *Thermocrinis* MAGs also encode the SOX system, which allows for the oxidation of thiosulfate to sulfate, as well as COX and cytochrome *c* oxidase allowing for respiration of O_2_. Additionally, the MAGs encode sulfide:quinone oxidoreductase, Sqr, allowing for oxidation of sulfide. As such, it is possible that *Thermocrinis* utilizes volcanic sulfide or thiosulfate (likely generated as a partial oxidation product of sulfide at alkaline pH (70)) as the electron donor and oxygen (O_2_) as the terminal electron acceptor to drive CO_2_ fixation, consistent with the high concentration of sulfide in the sample waters (Table 1). O_2_ is likely made available by ingassing of atmospheric gas, considering that the OFG aquifer waters are likely anoxic due to their high temperature and long inferred residence time in the subsurface (36). In addition to being able to use sulfide or thiosulfate as an electron donor, the *Thermocrinis* MAGs encode arsenite oxidase, AioAB, allowing the cells to oxidize arsenite as a source of reductant. Reduced arsenic species (e.g. arsenite) are particularly abundant in alkaline-chloride hot springs of YNP and are a consequence of water–rock interaction in the hydrothermal aquifer that is hosted in rhyolitic bedrock (71, 72). Thus, the capacity to use reduced arsenic species as an electron donor may be a unique adaptation of the dominant autotrophic populations (*Thermocrinis*) inhabiting geysers that are sourced by the deep hydrothermal aquifer in YNP.

The *Thermus aquaticus* MAGs from both the 2018 and 2019 metagenomes were also heterogenous and relatively incomplete, as observed for the *Thermocrinis* MAGs (Fig. 5). However, these MAGs exhibited high genomic similarity to the genome of *Thermus aquaticus*, suggesting the presence of multiple heterotrophic *Thermus* populations in the OFG communities (53). It is possible that *Thermus* is supported by chemosynthate or necromass produced by the dominant autotroph, *Thermocrinis*. Finally, the *Pyrobaculum* MAG in the 2018 metagenome was 92.0% complete with 55.8% estimated “contamination” due to the presence of multiple closely related populations (Fig. 5). *Pyrobaculum* in the 2019 metagenome showed the same general trend seen in the 2018 MAG: a relatively complete, but highly heterogenous MAG representative of multiple populations. The *Pyrobaculum* MAGs do not encode a complete hydroxypropionate–hydroxybutyrate carbon fixation cycle, as encoded by the most closely related strain *Pyrobaculum aerophilum* (73). Although it does not encode the entire pathway, three of the primary enzymes responsible for carbon fixation steps including pyruvate synthase and phosphoenolpyruvate carboxylase (73), in addition to 4-hydroxybutanoyl-CoA dehydratase, were encoded (74) (Table S5). Therefore, it is unclear whether the OFG *Pyrobaculum* population contributes to CO_2_ fixation. Nevertheless, the low relative abundances of *Pyrobaculum* (<1.0%) in the OFG communities suggest that they are unlikely to contribute significantly to community productivity, even if capable of CO_2_ fixation. The *Pyrobaculum* MAGs also encode most of the genes necessary for glycolysis, gluconeogenesis, and the TCA cycles, indicating the capacity for heterotrophy. Further, the MAGs encode nitrate reductases (NarGHI) and all the proteins necessary for dissimilatory sulfate reduction, including sulfate adenylyltransferase (Sat), adenylylsulfate transferase (AprAB), and dissimilatory sulfite reductase (DsrAB), indicating a potential to use nitrate or sulfate as terminal electron acceptors. These observations are consistent with the metabolic functions inferred in the type strain (75). Overall, the functional analyses of dominant MAGs support the hypothesis that OFG water communities are primarily dependent on chemolithoautotrophic metabolisms (mostly via dominant *Thermocrinis* populations), with few, low-abundance heterotrophs also present that likely are dependent on *Thermocrinis* for organic carbon production.

## Conclusions

Data presented here suggest the presence of an active subsurface microbiome inhabiting OFG that is largely supported by chemolithoautotrophic primary production. The similarity in ^14^CO_2_ fixation activities of vent and pool community members, along with the similarity in the taxonomic composition of their communities, suggests that the communities in the waters that collect in pools near the vent are seeded with active cells or, at a minimum, viable cells, by erupting OFG waters. At least two autotrophic populations are likely present in OFG waters based on different CO_2_ fixation activities at lower (70°C) and higher (90°C) temperatures. It is possible that these activities are attributable to different populations of *Thermocrinis*, of which two dominant populations and numerous additional subdominant populations were identified in both the pool and vent communities using 16S rRNA gene and metagenomic sequencing. Regardless, the short lag phase in CO_2_ fixation activity at 90°C when compared with 70°C suggests the presence of at least one autotrophic population that is adapted or acclimated to function better at a temperature similar to the vent (∼92–93°C).

The OFG pool contained high levels of *Thermocrinis* genomic diversity relative to YNP hot springs with similar geochemical profiles but that do not feature geysing. This genomic diversity may correspond to a collection of ecotypes, each exhibiting functional adaptations that are maintained through balancing selection to ensure function as their local environment fluctuates due to input of hotter water during periodic geysing events. These results suggest that the unique temperature (and presumably geochemical) dynamics in OFG, and geysers in general, may promote the generation and maintenance of higher levels of genetic diversity among closely related populations than more stable, nongeysing hot springs. High levels of genomic diversity among dominant *Thermocrinis*, *Thermus*, and *Pyrobaculum* populations that were also present in SG within YNP further support this hypothesis.

Envisioning a productive community comprising *Thermocrinis*, *Thermus*, *Pyrobaculum*, and other microorganisms in the subsurface aquifer waters of OFG is perhaps difficult, considering the temperature of those waters (110–118°C (14, 16)) and the extent to which they are supersaturated with CO_2_ that can be toxic to cells (22). Further, these waters would be expected to be limited in O_2_, given their long residence times in the subsurface aquifers sourcing OFG, a feature that is potentially inconsistent with the inferred aerobic metabolism of the dominant organisms, *Thermocrinis* and *Thermus*, identified in vent and pool waters. Perhaps more likely is that the walls of the conduit of OFG itself are amenable to colonization by microbial life, considering that the temperature of the conduit remains much more moderate (92–93°C) when compared with the shallow aquifer itself (110–118°C (14, 16)). Such an environment would still have access to water (steam), carbon (CO_2_), and chemical disequilibria (degassing of volcanic sulfide, infusion of atmospheric O_2_) capable of sustaining aerobic microbial metabolism. Thus, the detected populations in vent waters may be sloughed cells from biofilms on the walls of the vent that are physically removed during eruptive activity. Moreover, recent evidence has shown the presence of viable subsurface communities in YNP hydrothermal waters, including even at temperatures exceeding the boiling temperature of surface waters in YNP (76). Nevertheless, the discovery of a viable subsurface microbiome in OFG provides new insight into the habitability of these underexplored hydrothermal systems on Earth. Future studies are needed to assess whether the observations reported here can be generalized to other geyser systems, whether multiple closely related autotrophic populations exist in the OFG system due to differing temperature-dependent niches, and the extent of microbial activity within high-temperature subsurface waters. The traditional approaches used here (e.g. cell enumeration and activity assays) to detect life in geysed waters suggest that similar approaches could be used to detect life in geysers that exist on other planetary objects, such as the geyser plumes emitted from Enceladus. While cryovolcanic venting of fluids differs from the venting of fluids from OFG, methods to detect life in one fluid type to the other are likely applicable. Intriguingly, it has recently been suggested that cells (if present) should be emitted in geyser plumes from Enceladus (77), suggesting cell enumeration as a plausible strategy for life detection on this moon via a fly by mission.

## Materials and methods

### Sample collection and geochemical analysis

Old Faithful geyser (latitude: 44.460467, longitude: −110.828151) is in the Upper Geyser Basin in YNP, Wyoming, USA. Prior to sampling, the US Park Service provided researchers with information to ensure safe sampling under research permit YELL-SCI-5544. They then escorted researchers onto the cone of OFG to ensure compliance with these safety protocols. Samples for geochemical and microbiological measurements were taken from the splash pool, referred to herein as pool water, adjacent to the vent of OFG on 2018 November 9, 2019 November 8, and 2020 November 5, during the administrative period when YNP is closed to visitors in motor vehicles. Samples for geochemical and biological measurements were also taken from the erupted (geysed) water, referred to as vent water, on 2019 November 8 and 2020 November 5. The vent water was collected by catching it in a presterilized plastic bin (described below) during an eruption and pool water was collected using a peristaltic pump (described below). An Easy Log EL-USB-TC Type K (Lascar Electronics, Erie, PA, USA) thermocouple was placed in the splash pool prior to an eruption on 2019 November 8, to log temperature changes in the pool. Samples of water were also collected from another geyser within the Upper Geyser Basin, Spouter Geyser (SG; latitude: 44.463062, longitude −110.853030) on 2018 November 9, for geochemical and genomic analyses to facilitate comparison with OFG.

At the laboratory and prior to sampling, a 20-L plastic tub (high-density polyethylene) was washed with 10% bleach three times and rinsed with MilliQ water. The bin was then autoclaved in an autoclave bag with the lid in place. The bag was sealed after autoclaving and transported to OFG and placed on the cone for collection of the vent water. To keep the bin in place as erupted waters were being collected, two 8-pound weights were washed with bleach three times, rinsed with MilliQ water, wrapped in sterile autoclaved aluminum foil, and then placed in the bin prior to the eruption. The bin and weights were left on the cone for the entirety of the eruption cycle. At OFG, roughly 10 min following an eruption, water was pumped directly from the pool and the plastic bin containing vent water through tubing for ∼5 min to flush it, using a field deployable peristaltic pump (GeoTech, Denver, CO, USA). Vent waters were collected first, followed by pool waters. A subsample of vent and pool water was collected for carbon fixation activity assays, cell counts, and DNA extraction (described below). Subsamples of water were also collected for geochemical measurements, including field determinations of pH and temperature using a WTW combination pH probe (Xylem, Rye Brook, NY, USA) and conductivity with a temperature-compensated YSI meter (YSI Inc., Yellow Springs, OH, USA). Total sulfide (H_2_S, HS^−^, plus acid labile metal sulfides; termed, total S^2−^) and ferrous iron (Fe(II)) concentrations were quantified from nonfiltered water using Hach sulfide reagents 1 and 2, Hach ferrozine pillows, and a Hach DR/890 field deployable spectrophotometer (Hach Company, Loveland, CO, USA). OFG samples for anion, cation, trace metals, and water isotopes were filtered (0.22 µm) on site, placed on ice, acidified (trace metals), and immediately sent to the Montana Bureau of Mines and Geology for the analysis of water composition using ion chromatography, inductively coupled plasma (ICP)-optical emission spectroscopy, ICP-mass spectrometry, and water isotope analysis (Table S6). Water samples were also collected for dissolved organic carbon (DOC) determination by filtering water (0.22 µm) into a glass vial. Samples were stored on ice in the dark until returned to the laboratory and at 4°C in the dark until analyzed at the Environmental Analytical Laboratory at Montana State University using a Shimadzu total organic carbon analyzer. Waters for determining SO_4_^2−^ and Cl^−^ concentrations in 2018 were filtered on site (0.22 µm) into acid-washed polypropylene vials and stored at 4°C. Concentrations of SO_4_^2−^ and Cl^−^ were measured with Hach reagent kits and a spectrophotometer upon return to the laboratory. SG samples for DNA extraction and geochemical analysis were collected as described for the OFG samples.

### Cell counts

Cell density was determined in both the pool and vent waters collected in 2020 using fluorescent microscopy. Ten milliliters of water was subsampled in triplicate with a sterile syringe and transferred to sterile 15 mL tubes containing formaldehyde to a final concentration of 2% *v*/*v*. At the laboratory, 1.5 µL of SYBR Gold (100× stock solution) and 2 µL of DAPI (2 mg mL^−1^) were added to each 10 mL water sample and these were incubated in the dark at room temperature for 20 min. Stained cells were collected on 0.22 µm black polycarbonate filters (Millipore, Billerica, MA, USA) and were visualized using an EVOS fluorescent microscope (Life Technologies, Carlsbad, CA, USA). DAPI- and SYBR Gold–stained cells were imaged, and images of filtered cells were overlayed using the “overlay” function in the EVOS software package to better differentiate debris and minerals in the water from cells. Thirty images were randomly captured, and cell counts for each water type (pool and vent) were performed in triplicate.

### Activity assays

Samples from the vent and the pool waters collected in 2020 were used to measure rates of ^14^C-bicarbonate assimilation into biomass using methods previously described (78, 79). Roughly 1 L of both the vent and pool water was collected within 10 min of an eruption and taken to the boardwalk on the south side of OFG to set up activity assays. Ten milliliters of water was distributed into capped and autoclaved 24 mL serum vials using a sterile needle and syringe. Abiotic assays were also set up as described above; however, the water was filtered (0.1 µm) as it was being injected into the vials. Once all the water was aliquoted, the 24 mL serum vials and their contents were stored on ice for transport back to the laboratory. At the laboratory, biotic and 0.22 µm filtered (abiotic) control microcosms were spiked with 7.5 µCi of ^14^C-labeled sodium bicarbonate (NaH^14^CO_3_) and were incubated in the dark at 70 and 90°C for 2, 4, 8, and 16 h. Assays were prepared in triplicate for each condition (biotic and abiotic), incubation temperature, and incubation length. Following the specified incubation times, assays were acidified by the addition of 12 N HCl to a pH < 2.0, allowed to degas for 2 h in a fume hood, and then filtered onto 0.2 µm white nylon membrane filters (GE Healthcare, Chicago, IL, USA). The filters were then overlayed with 10 mL of Cytoscint ES scintillation cocktail and subjected to liquid scintillation counting using a PerkinElmer Tri Carb 2900TR Liquid Scintillation Analyzer (PerkinElmer, Waltham, MA, USA). Data are reported in counts per minute (CPM) normalized to volume of sample in each microcosm. It is important to note that the activities measured in the vent waters and pool waters are not directly comparable, due to the vent waters likely being supersaturated with CO_2_ since a buildup of this gas is thought to, at least in part, drive eruptive behavior (7, 22). In contrast, the waters in the splash pool presumably outgassed some CO_2_ between eruptions. This would result in a lower fraction of labeled to unlabeled DOC in the microcosms containing vent water, which may lead to lower observed rates of ^14^CO_2_ fixation activity. Due to time limitations while sampling on the cone of OFG, dissolved gas concentrations for the waters were not determined.

### DNA extraction

Samples for DNA extraction and sequencing were collected in 2018, 2019, and 2020, at the same time as geochemical measurements were made, ∼10 min after eruption activity dissipated at OFG. Samples were also collected in 2018 from Spouter Geyser (SG) spanning eruptive and inactive periods. In the case of SG, water was collected directly from a pool below the main geyser that fills and drains during an active eruption. For OFG, water was pumped directly from the pool and the plastic bin containing vent water using a field deployable peristaltic pump to flush the tubing. After flushing, a presterilized aluminum filter housing (Pall Corporation, Port Washington, NY), containing a sterile 45 mm-diameter, 0.22 µm pore size polycarbonate filter (Pall Corporation, Port Washington, NY), was placed at the end of the tubing and used to collect planktonic biomass from ∼4 L of water. After sampling pool waters, ∼4 L of vent water was filtered using the same approach. Filters were aseptically removed from the aluminum housing, placed in sterile 50 mL Falcon tubes, and frozen on site using dry ice for transport back to the laboratory, where they were stored at −80°C until further processed. Samples from the vent water for DNA extraction were not collected in 2018. Prior to DNA extraction and in a UV pretreated laminar flow hood, filters were placed in a sterile petri dish and were cut in half using a sterile scalpel. DNA was extracted from filters using the MP Bio FastDNA spin kit and the concentration of DNA in each extract was quantified using the Qubit dsDNA HS assay kit and fluorometer. Equal volumes of two replicate extracts were pooled for metagenomic sequencing.

### 16S rRNA gene amplification, sequencing, and analysis

DNA collected from filtered biomass from OFG in 2020 was subjected to triplicate PCR amplification of the 16S rRNA gene using the modified universal primers 515F (5′-GTGYCAGCMGCCGCGGTAA-3′) and 806R (5′-GGACTACNVGGGTWTCTAAT-3′) according to previously described methods (80). PCR amplification was conducted in triplicate for each sample, and amplification was verified via gel electrophoresis. Equal volumes of triplicate amplicons were pooled for each sample. Sequence adapters were then added to PCR amplicons using 5 μL of product and eight cycles of PCR at an annealing temperature of 50°C. The amplicons were then sequenced by the University of Wisconsin Biotechnology Center Sequencing Facility (UWBCS) using paired-end sequencing (2 × 300 bp) on the Illumina MiSeq platform.

16S rRNA gene sequences were analyzed and processed using Mothur (ver. 1.43.0) (81) with a previously developed MiSeq standard operating procedure (82). Briefly, sequences and quality scores were extracted, and merged paired end reads were made into contigs using the make.contigs command. The reads were then screened and quality filtered using the screen.seqs command to reduce sequence redundancy and errors. Unique sequences were then identified and aligned to the SILVA database (v132) (83). Operational taxonomic units (OTUs) were assigned at a nucleotide sequence similarity of 97.0%. Finally, OTUs were taxonomically classified using the Bayesian classifier of Mothur (84) (Table S2) and the taxonomic classifications were manually verified with BLASTn.

16S rRNA gene phylogenetic analyses of the dominant OFG water populations (*Thermocrinis* sp.) were conducted by searching for other closely related references (>∼94% nucleotide identity) within the National Center for Biotechnology Information (NCBI) and Integrated Microbial Genomes databases. Related references were compiled with the representative sequences for the *Thermocrinis* OTUs and aligned in mothur v.1.45.2 (81) against the SILVA v138 alignment template. The alignments were also filtered in mothur and then subjected to Maximum Likelihood analysis in IQTREE v.1.6.12 (85) after identifying the optimal nucleotide substitution model (TIM3+F+R3) based on the “TEST” model selection algorithm. The final 16S rRNA gene tree was chosen from ten independent separate runs, and node support was evaluated with 1,000 ultrafast bootstrap replicates. Metadata (environmental source, hot spring temperature, and hot spring pH) was collected for the reference 16S rRNA genes, where these data were available (Table S3).

### Metagenomic sequencing and analysis

Genomic DNA collected in 2018 and 2019 from pool samples in OFG and SG (2018 only) were subjected to library preparation and shotgun sequencing at UWBCS using the Illumina NovaSeq6000 platform (2 × 150 base pair sequencing). Due to low genomic DNA yields, library preparations for DNA collected from vent water in 2019 and 2020 were unsuccessful. Reads were trimmed and downsampled with the TrimGalore v.0.6.0 and BBMap programs to cleave sequencing adapters and remove sequencing redundancies as previously described (65). Trimmed and downsampled sequences from 2018 and 2019 were assembled individually and coassembled using Spades v.3.14.0 specifying default parameters. The quality of the assemblies was then compared using various assembly metrics and the metaquast program (v.4.3) (86). The coassembled metagenomes resulted in substantially higher quality metagenome assemblies, and these were thus further used to characterize the OFG pool communities. Assembled contigs were binned into MAGs using MetaBAT v.0.26.3 (87), based on read depth and tetranucleotide frequency specifying the “verysensitive” setting. Both the 2018 and the 2019 assemblies were binned separately after coassembly, and the quality, completeness, and level of contamination of each bin were assessed using CheckM v.1.0.5 (88). “Outlier” contigs were removed with RefineM v.0.0.23 (89). CheckM v.1.0.5 was used to reassess the binning of the 2018 and 2019 metagenomes after these modification steps were complete.

Gene predictions and annotation of genome bins were made using PROKKA v 1.11 (90). These protein annotated files were then uploaded to the Kyoto Encyclopedia of Genes and Genomes (KEGG) database (91) using the KEGG Automatic Annotation Server (92) to further examine the potential functionalities encoded by MAGs. MAGs were also examined for key functionalities related to chemolithoautotrophic primary production (e.g. hydrogen metabolism, sulfur metabolism, and carbon fixation) using BLASTp and protein queries from cultured representatives with those demonstrated capabilities (see Table S5), as described previously (48). Briefly, BLASTp results were filtered using an *E*-value cutoff of 10*e*^−30^ and query cover of >50% amino acid identity. The presence or absence of proteins of interest within *Thermocrinis* and *Pyrobaculum* bins is listed in Table S5. The relative abundances of dominant populations (>∼1% relative abundance) were estimated based on mapping of quality-filtered reads from the metagenomes to the Kraken2 database (93) using the MetaWRAP pipeline (94).

To assess the hypothesis that the temporal dynamics of geysing activity selects for greater diversity of microbial communities, the strain-level diversity of dominant populations (*Thermocrinis*, *Thermus*, and *Pyrobaculum*) was compared among the geyser water samples analyzed here and from waters and sediments from other YNP hot springs. Specifically, the OFG 2018, OFG 2019, and SG 2018 metagenomes were compared against those for other circumneutral (range of spring pH: 5.2–7.2), high-temperature (range of spring temperatures: 76.5–85.9°C) hot springs of YNP where these populations have previously been observed at >1% relative abundance and where sequencing data was of sufficient depth and quality to permit such comparisons (Table S3). Despite multiple attempts and strategies to recover high-quality *Thermocrinis* MAGs from the OFG and SG waters, only fragmented, highly heterogeneous MAGs could be generated. Thus, to objectively assess strain-level *Thermocrinis* diversity across systems, nucleotide diversity metrics of *Thermocrinis* (and *Thermus* and *Pyrobaculum*)-mapped reads were compared among metagenomes (described in more detail below). Identification of RNA polymerase subunit beta (RpoB) encoding genes within the geyser metagenomes and comparison against the NCBI nonredundant (nr) database revealed high amino acid identity of the geyser *Thermocrinis* RpoB sequence to that of the type strain, *T. ruber* (e.g. 94–99% identity). Similarly, the OFG *Thermus* and *Pyrobaculum* RpoB sequences exhibited high amino acid identities with *Thermus aquaticus* and *P. yellowstonensis*. Consequently, complete genomes from *T. ruber* (NCBI accession: CP007028), *Thermus aquaticus* (NCBI accession: CP010822), and *P. yellowstonensis* (NCBI accession: PRJNA258558) were used to map metagenomic reads from the OFG 2018, OFG 2019, and SG 2018 metagenomes, in addition to those from other hot springs (Table S3) using bowtie2 v. 2.3.3.1 (95). The relative abundances of the *Thermocrinis*, *Pyrobaculum*, and *Thermus* populations were also assessed among the metagenomes identified above and those from waters and sediments generated in our other studies (Table S3). Relative abundances were calculated as the summed percentage of reads in each metagenome identified as one of the three genera, as assessed with read mapping against the Kraken2 database.

The nucleotide diversity was then determined among the mapped reads using the “profile” function of inStrain v.1.5.5 (96) with default parameters. Average nucleotide identity (ANI) values of 95 and 90% were initially used as threshold cutoffs for filtering mapped reads. Nearly identical results were observed for both *Thermocrinis* and *Thermus*, and thus, the reported nucleotide diversity metrics were quantified from 95% ANI-filtered reads. In contrast, the 90% ANI-filtered reads for *Pyrobaculum* captured considerably more diversity than 95% ANI filtering, and thus, the former was used for the *Pyrobaculum* populations only. Nucleotide diversity (calculated as 1−[(*A*_frequency_)^2^ + (*C*_frequency_)^2^ + (*T*_frequency_)^2^ + (*G*_frequency_)^2^] and SNV counts are reported for the *T. ruber*-, *T. aquaticus*-, and *P. yellowstonensis*-associated reads from each metagenome.

To further evaluate the phylogenetic diversity of the three above populations in context of other hot springs systems in YNP, the RpoB subunits within each metagenome (among both genome-binned and nonbinned contigs) were identified by BLASTp searches of the *T. ruber*, *T. aquaticus*, and *P. yellowstonensis* RpoB proteins against protein coding gene annotations for the above hot springs or OFG metagenomes, in addition to publicly available metagenomes from YNP. Complete and partial RpoB subunits were then aligned with *Thermocrinis*, *Thermus*, and *Pyrobaculum* reference sequences from the NCBI nr database using Clustal omega (97) and subjected to maximum likelihood phylogenetic analysis in IQ-TREE v.1.6.12 (85) after identification of the optimal amino acid substitution model using the ModelFinder Plus model selection tool. Prior to ML analysis, short partial RpoB sequences were removed by trimming with trimal (98) and removal of sequences containing <50% of overlapping residues in the alignment (-resoverlap 0.5 and -seqoverlap 50). Ultrafast bootstraps (1,000 replicates) were used to evaluate the node support for the resulting phylogenetic reconstruction.

## Supplementary Material

pgad066_Supplementary_Data

## Data Availability

All sequencing data produced by this study are available under NCBI bio project accession number PRJNA854645. All geochemical data produced by this study are available in the tables and supplemental tables provided.
