# Supplementary material for: An active microbiome in Old Faithful geyser
Source: PNAS Nexus. 2023 Mar 4;2(3):pgad066. doi: 10.1093/pnasnexus/pgad066 (PMC10062350; doi:10.1093/pnasnexus/pgad066)
Supplement: pgad066_Supplementary_Data [file pgad066_supplementary_data.zip › PNASNEXUS-PNASNEXUS-2022-01292-T-s01.docx]

**
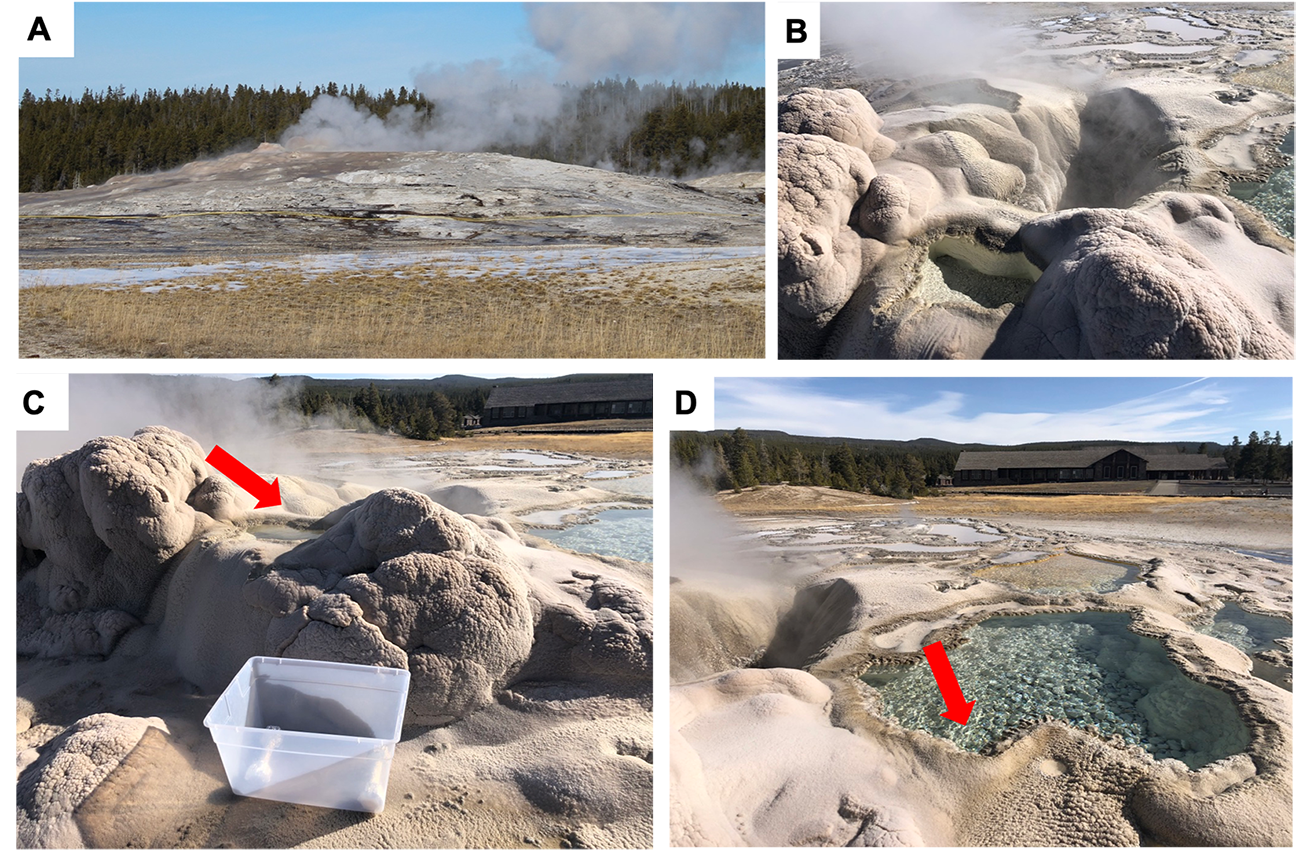
**

**Figure S1. Images of Old Faithful Geyser and the pool where samples for chemical and biological characterization were collected. A.** Cone of Old Faithful from a distance, showing the platform of siliceous sinter that has been deposited over several hundred to thousands of years. **B.** Image looking eastward of the conduit of Old Faithful, with splash pools in the background of this image. **C.** Image of the sterilized catch tub adjacent to the cone of Old Faithful that was used to collect plume waters. The arrow is pointing to the opening of the conduit of Old Faithful. **D.** Location of the splash pool where the samples were collected (red arrow) relative to the primary vent for OF. Arrow depicts the location where water samples were collected and where the thermocouple was placed during the course of an eruption cycle


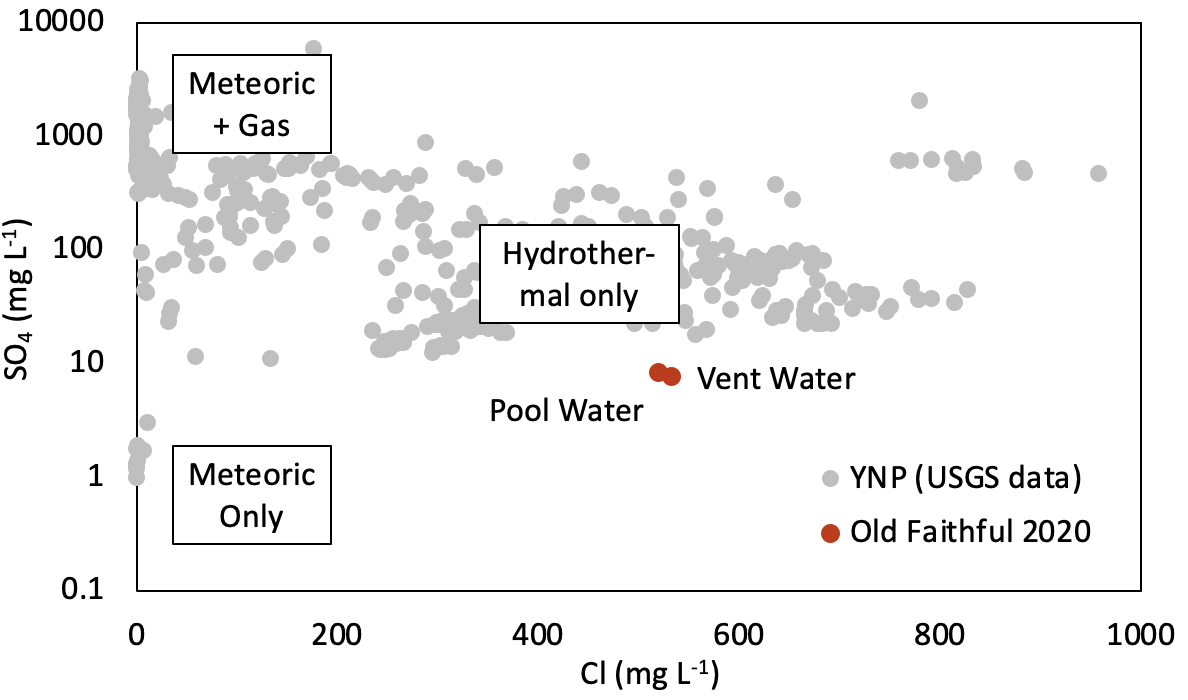


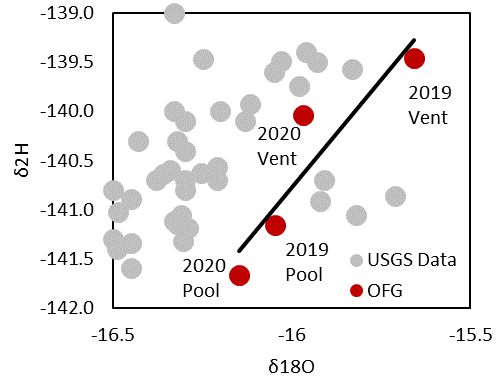

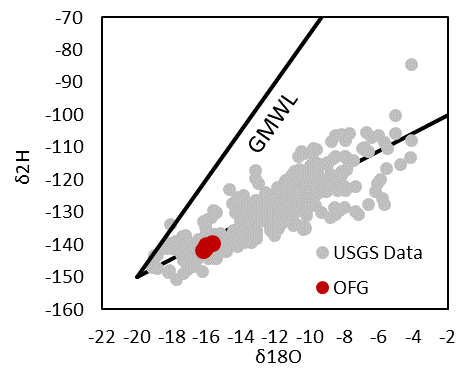


**B**

**C**

**A**

**Figure S2. Distribution of sulfate and chloride concentrations and water isotopes of hot springs in YNP. A)** Plot of sulfate (SO_4_^2-^) and chloride (Cl^-^) values collected from Yellowstone National Park (YNP) hot springs between 2003-2013 from previously published United States Geological Survey (USGS) data shown in grey (1-3) in context of end-fluid member classifications that have been previously described (4). The SO_4_^2-^ and Cl^-^ concentrations of the Old Faithful Geyser vent and splash pool waters are indicated in red. **B)** Water isotope values collected from YNP hot springs from previous USGS published data (1-3)is shown in gray. OFG data points are represented in red. YNP data is plotted against the GMWL (global water meteoric line). **C)**  Close up view of OFG data. OFG data is represented by red data points.


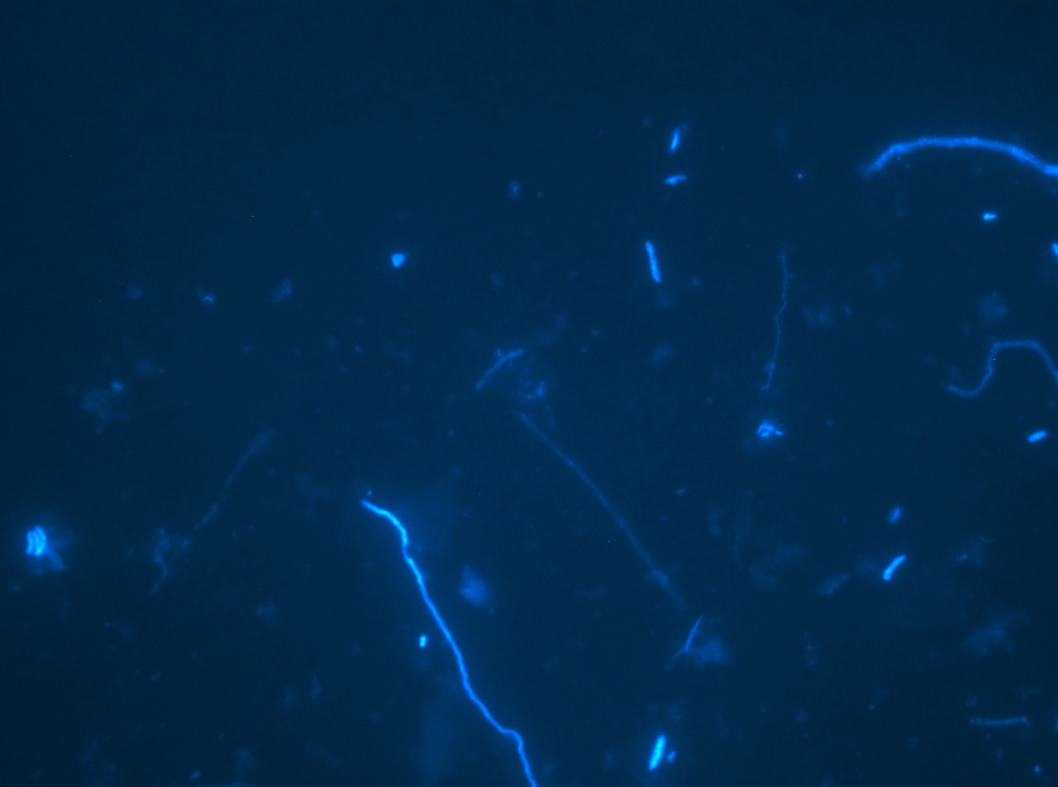


10 **μm**

**A**


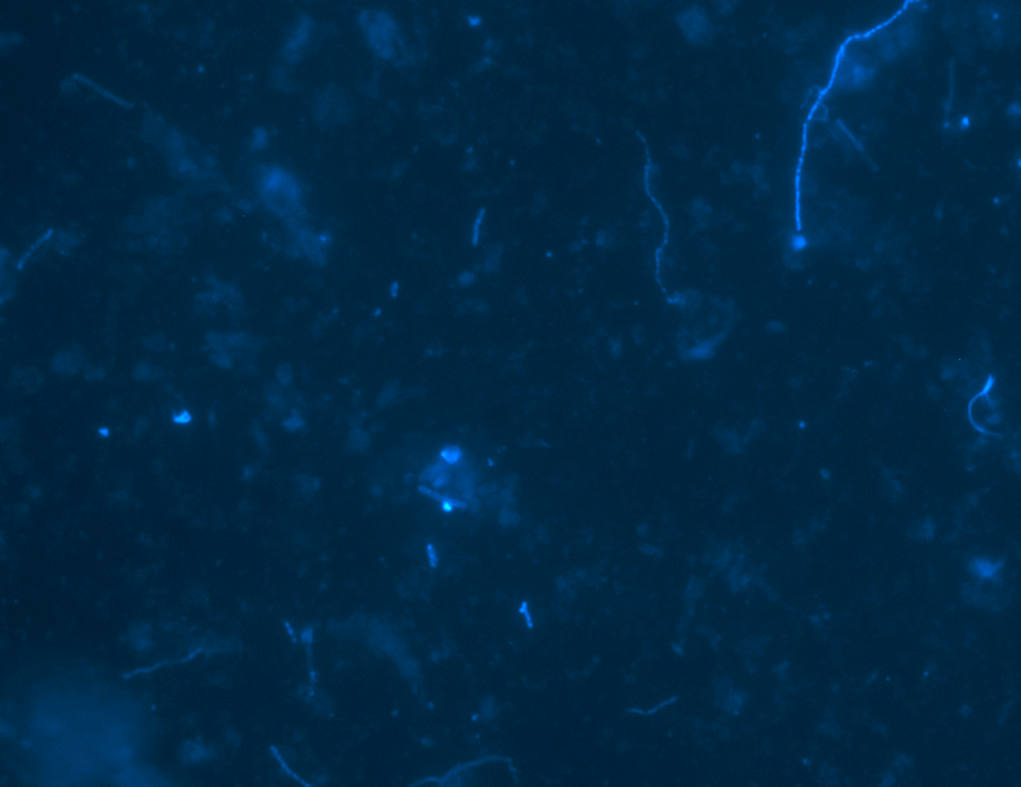


10 **μm**

**B**

**Figure S3. Fluorescent microscope images of water from the Old Faithful Geyser plume and splash pool. A.** DAPI-stained cells from the splash pool water samples imaged with a 100x objective. **B.** DAPI-stained cells from plume waters imaged with a 100x objective. Scale bars showing sizes are indicated in the bottom left.


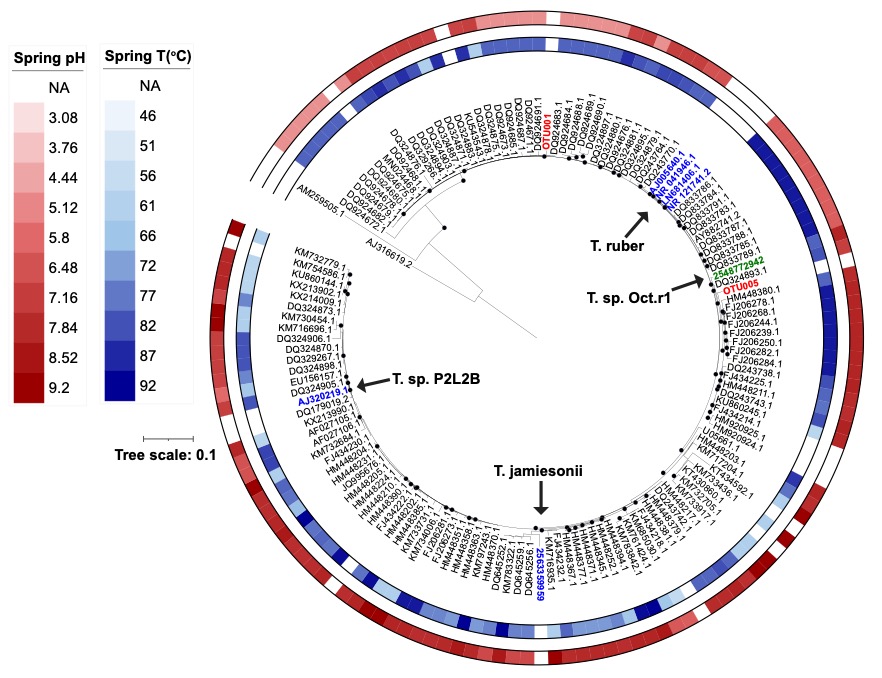


**Figure S4. Phylogenetic reconstruction of predominant *Thermocrinis* 16S rRNA genes recovered from OFG waters.** A Maximum Likelihood reconstruction is shown including representative sequences from the two predominant OTUs recovered from 16S rRNA gene analysis of OFG waters (OTU001 and OTU005; highlighted in red). In addition, 16S rRNA gene sequences from previously published studies were included based on BLAST searches of the two OFG OTUs against the NCBI and IMG databases. Cultured representatives are highlighted with arrows and their accessions are bolded in blue. The accession for the single metagenome-assembled-genome (MAG) represented in the tree is shown in bolded green text. Black circles indicate bootstrap values >50% (out of 1,000 bootstrap replicates). Outgroup sequences for *Hydrogenobaculum* sp. Y04ANC1 (AM259505.1) and *Thermovibrio rubrum* strain DSM14644 (AJ316619.2) are also included. Branch length is scaled according to the scale on the left showing the expected number of substitutions per site. The temperature and pH of the hydrothermal water where sequences derive from are shown by the blue and red scales on the left and in the inner and outer rings, respectively. Where data were not available or the sequences did not derive from a hydrothermal environment, white blocks are shown in the rings. The data and associated references for the sequences are provided in **Table S1**. Note that the placement of the two OTUs is only based on ~250 bp.

**
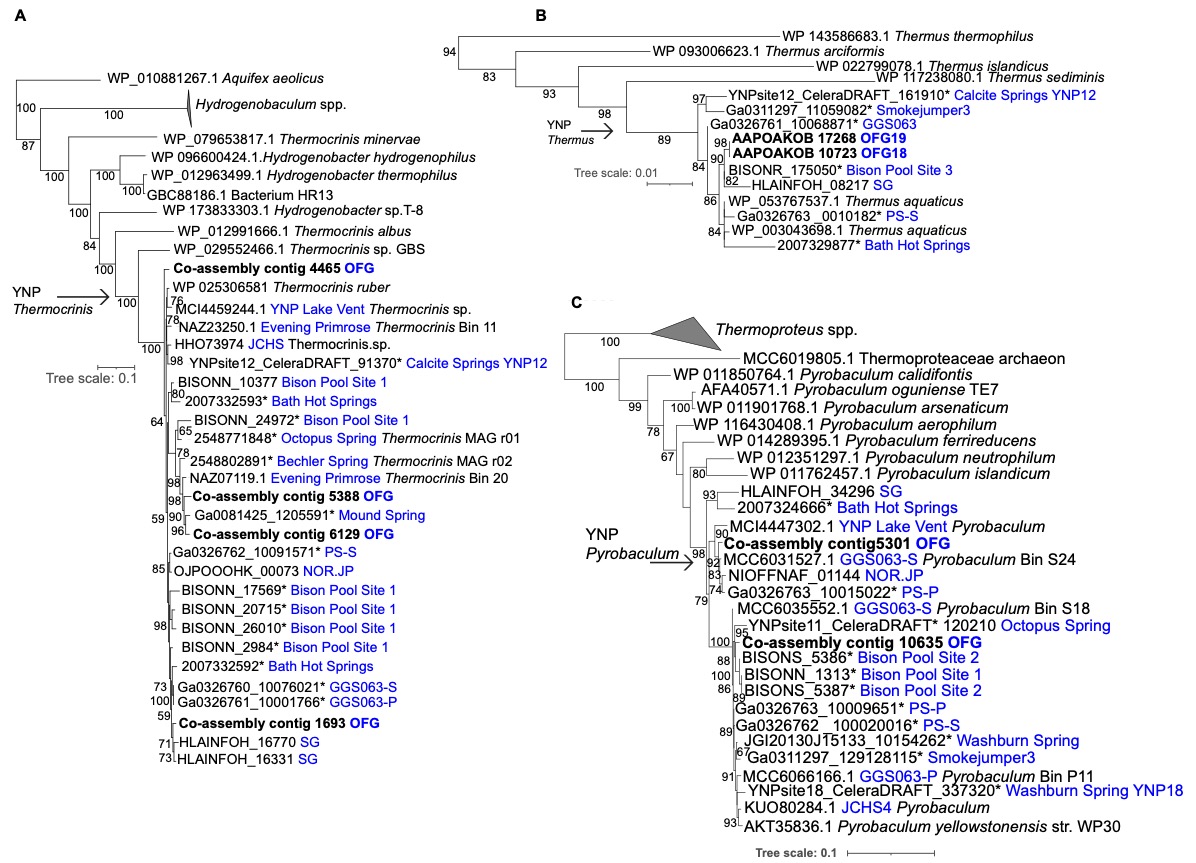
**

**Figure S5. Phylogenetic reconstruction of RNA polymerase subunit beta (RpoB) proteins encoded by A) *Thermocrinis*, B) *Thermus*, and C) *Pyrobaculum* phylotypes.** Maximum-Likelihood phylogenetic reconstructions were constructed using RNA polymerase subunit beta (RpoB) proteins encoded in the OFG and SG assemblies, in addition to other YNP hot spring water (plankton) or sediment communities, and closely related reference genomes in the NCBI or JGI IMG databases. The *Thermocrinis* and *Pyrobaculum* proteins were encoded in the 2018 and 2019 OFG co-assembly, while *Thermus* RpoB were not detected in the co-assembled contigs, but were identified in the individual 2018 and 2019 assemblies. Consequently, the two *Thermus* RpoB proteins from the 2018 and 2019 are shown. The RpoB alignments comprises 1,530, 1,119, and 1,148 positions, respectively. Bootstrap values (out of 1,000 bootstraps) are shown at the branches where >50. The scale bar next to each reconstruction shows the expected number of substitutions per site for that phylogeny. Bolded entries are from the OFG assemblies. YNP-group designations indicate monophyletic groups comprising RpoB from YNP hot spring metagenomes, including those that were the focus of this study (or isolates also recovered from YNP springs). Sequence identifiers show accession number (with an asterisk if from the IMG database), followed by the isolate or sequence ID. The YNP spring that the sequence derives from is highlighted in blue.

**Figure S6. Distribution of *Thermus* and *Pyrobaculum* across Yellowstone National Park (YNP) hot spring and geyser communities and their association with genomic nucleotide diversity. A, C)** Relative abundances of *Thermus* (**A**) and *Pyrobaculum* (**C**) populations in 35 hot spring and geyser communities (**Table S4**). Each point represents one metagenome that is plotted based on spring temperature and pH. Metagenomes with <1% of reads are shown as grey squares (sediment metagenomes) or circles (water metagenomes), while those with populations comprising >1% of mapped reads are colored according to the scale to the right of the plots (note that the relative abundance scales differ from each other and that in Figure 6. **B, D)** Genomic nucleotide diversity of *Thermus*-like (**B**) and *Pyrobaculum-*like (**D**) populations (based on >95% ANI mapping to the *Thermus aquaticus* genome and >90% ANI mapping to the *Pyrobaculum yellowstonensis* genome) as a function of population relative abundances within metagenomes. Non-geysing hot springs (n=10) are shown as grey circles and geysers are shown as black circles.

**References**

1. J. W. Ball, R. B. McCleskey, D. K. Nordstrom (Water-chemistry data for selected springs, geysers, and streams in Yellowstone National Park, Wyoming, 2003-2005. (U. S. Geological Survey Open-File Report 2010-1192 Reston, VA 2010).

2. J. W. Ball, R. B. McMleskey, D. K. Nordstrom (Water-chemistry data for selected springs, geysers, and streams in Yellowstone National Park, Wyoming, 2006-2008. (U. S. Geological Survey Open-File Report 2010-1192 Reston, VA 2010).

3. R. B. McCleskey, Chiu, R.B., Nordstrom, D.K., Campbell, K.M., Roth, D.A., Ball, J.W., Plowman, T.I. (Water-chemistry data for selected springs, geysers, and streams in Yellowstone National Park, Wyoming, beginning 2009. (Geological Survey Online Database, 2014).

4. D. K. Nordstrom, R. B. McCleskey, J. W. Ball, Sulfur geochemistry of hydrothermal waters in Yellowstone National Park: IV Acid-sulfate waters. *Appl. Geochem.* **24**, 191-207 (2009).
